# Low enhancing papillary renal cell carcinoma diagnosed by using dual energy computerized tomography: a case report and review of literature

**DOI:** 10.1186/1471-2490-14-102

**Published:** 2014-12-19

**Authors:** Shaheen Alanee, Danuta I Dynda, Patrick Hemmer, Bradley Schwartz

**Affiliations:** Southern Illinois University School of Medicine, Division of Urology, 301 N Eighth St - St John’s Pavilion, PO Box 19665, 62794-9665 Springfield, IL USA; Center for Clinical Research/Division of Urology, Southern Illinois University School of Medicine, 301 N Eighth St - St John’s Pavilion, PO Box 19665, 62794-9665 Springfield, IL USA; Laboratory Medical Director, Saint John’s Hospital, 800 East Carpenter Street, 62769 Springfield, IL USA

**Keywords:** Papillary renal cell carcinoma, Renal cyst, Dual energy computerized tomography

## Abstract

**Background:**

Papillary renal cell carcinoma (pRCC) is a mixed group of tumors that constitutes about 15-20% of all renal cortical cancers. Strong enhancement on computerized tomography (CT) is a feature of clear cell, but not of pRCC making the differentiation of papillary tumors from benign cysts a diagnostic problem in some cases.

**Case presentation:**

We report here a case of a female patient with pRCC that was initially diagnosed as a benign renal cyst. The patient is a 66 year old Caucasian female who initially presented with an ultrasound showing a 2.6 cm hypo-echoic lesion within the inferior pole of her left kidney. This was followed by a contrast enhanced computerized tomography that suggested the hypo-echoic lesion to be a hyper-attenuating benign renal cyst. Follow-up CT scan 4 months later demonstrated an increase in the size of the lesion to 3.2 cm with equivocal enhancement. A dual energy computerized tomography (DECT) showed the lesion to be a solid mass suspicious for renal cell carcinoma. A robotic partial nephrectomy revealed a papillary renal cell carcinoma with negative margins.

**Conclusion:**

In this case report, we reviewed the literature on variations in enhancement of renal tumors and the possible role of dual energy contract enhanced CT in differentiating papillary tumors with low enhancement from benign kidney cystic lesions.

**Electronic supplementary material:**

The online version of this article (doi:10.1186/1471-2490-14-102) contains supplementary material, which is available to authorized users.

## Background

Papillary renal cell carcinoma (pRCC) is the second most common type of renal tumors accounting for 15-20% of kidney cancer [1, 2]. pRCC is divided into type I and type II differentiated on the basis of cytological and architectural features [3–5]. These tumors tend to show low enhancement on computerized tomography (CT) imaging posing a diagnostic dilemma for the practicing physician. We describe a case of papillary renal cell carcinoma that was initially diagnosed on contrast enhanced CT as a benign cyst, with the discussion of how dual energy CT (DECT) scan could be helpful in making the right diagnosis in such situations.

## Case presentation

**Figure 1:**
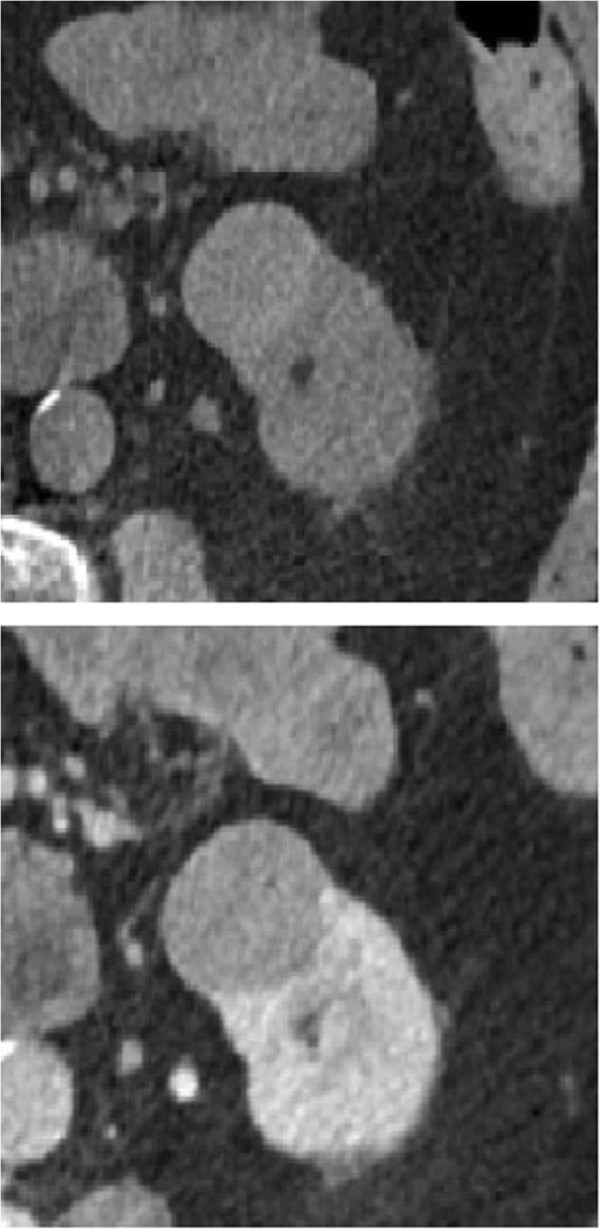
**Radiological imaging of the patient's kidney tumor (a) (top): Unenhanced CT of kidney and tumor.** **(b)** (bottom): Enhanced CT of kidney and tumor.

A 66 year-old Caucasian female, with past medical history significant for asthma, presented with an asymptomatic 3.2 cm lesion within the inferior pole of the left kidney. The patient had no family history of kidney cancer and there were no significant findings on examination. The lesion had been diagnosed a few months ago on an abdominal ultrasound as a hypo echoic 2.6 circular abnormality. No significant vascularity was seen in the lesion using doppler ultrasound. A follow-up contrast enhanced CT showed the abnormality to have Hounsfield attenuation units of 63. The attenuation increased to 70 units on the early arterial and delayed phases. A diagnosis of a hyper-attenuating benign renal cyst was consequently made. A follow-up CT scan showed an increase in the size of the lesion to 3.2 cm within 4 months and to have equivocal enhancement of about 10 Hounsfield units on post contrast images. The lesion did not demonstrate significant wall thickening, septations, or mural nodules (Figure 1a). The patient then presented to our clinic for further management. Fortunately, a DECT scanner was recently installed at an affiliate hospital of the Southern Illinois University School of Medicine (St John’s Hospital - Springfield, IL), and the Radiology Department at St. John’s Hospital had communicated to us the possible benefit of DECT in renal tumors with equivocal enhancement characteristics. A shared decision was made with the patient to proceed with DECT imaging of the kidneys to further characterize her renal lesion. DECT was performed using Siemens 128 dual source dual energy computerized tomography machine, and Siemens Syngo Via software was used to process the images. Iodine was noticed inside the lesion on DECT indicating tissue enhancement that is associated with solid tumors (Figure 1b). Recommendation was made to the patient for tumor removal and surgery was done robotically with no complications. Intra-operatively, the lesion was found to be a solid tumor emanating from the lower pole of the left kidney (see Additional file 1 for timeline).

### Pathologic results

**Figure 2:**
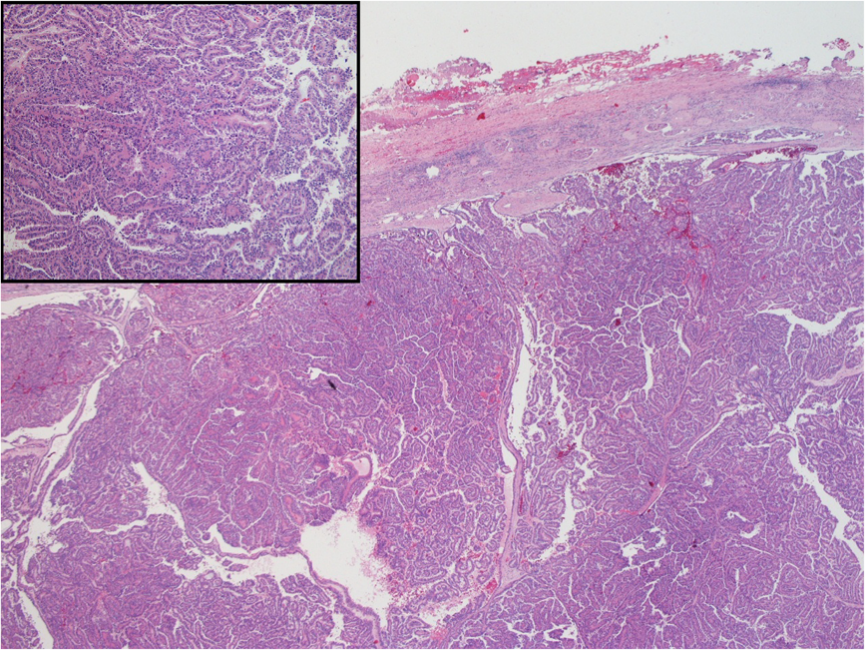
**Hematoxylin and Eosin staining of the tumor tissue showing characteristic histology of papillary renal cell carcinoma.** (inset: 100× magnification).

The final pathology demonstrated papillary renal cell carcinoma type I, approximately 3 cm in size, confined to the kidney with negative surgical margins (Figure 2).

## Discussion

The incidence of RCC is rising worldwide, with RCC currently accounting for almost 3% of solid malignant tumors [6]. Obesity, and increasing utilization of imaging modalities are some of the factors contributing to the increase in RCC incidence [7–9]. CT scan is now recognized as the gold standard for evaluating RCC because it provides information about the tumor itself and its extension to surrounding structures. Multiple previous studies have attempted to differentiate clear cell carcinoma from other subtypes of the same disease using enhancement qualities, however, the only solid finding was that strong enhancement is a unique finding for clear cell RCC [10, 11]. The diagnosis of papillary renal cell carcinoma is specifically problematic on CT since pRCC tend to enhance to a lesser extent than the normal renal cortex, and could be confused with more benign kidney lesions such as in the case of the patient we present in this report. The low vascularity of pRCC could be used to explain the low enhancement of these tumors making their differentiation from benign kidney cysts problematic. In fact, it was not unusual for pRCC to show no enhancement on angiography, when that modality was used to diagnose RCC before CT dominated the field. In addition, while clear cell tumors are generally large and have areas of necrosis, hemorrhage, and calcification, pRCC are generally smaller and homogeneous adding to the difficulty of differentiating them from fluid filled renal cysts.

Dual energy CT scan is a recent development in imaging technology that could change the way we image and follow-up on renal tumors [12]. DECT deals with many of the drawbacks of the contrast enhanced multiphase CT that we currently use to manage kidney tumors. DECT decreases the amount of radiation patients are exposed to due to the decrease in number of phases needed for a renal CT scan because it depends on one phase therefore omitting the need for pre-contrast images [13–16]. Furthermore, recent advances in DECT is allowing for synthesis of monochromatic images from polychromatic data through spectral separation, which promises a more accurate measurement of renal tumors enhancement [17–19]. DECT works on simultaneous acquisition of two different energy spectra, and thus can acquire accurate information about tissue composition. DECT uses photoelectric energy emitted when an incident photon causes an electron to be emitted from atomic shells, an event common for elements with high atomic number like Calcium and Iodine, to identify different compounds. Therefore, DECT is able to differentiate between iodine and the surrounding soft tissue based on the difference in photoelectric energy between Iodine and the elements constituting human soft tissue (oxygen, carbon, nitrogen, and hydrogen) [20].

Iodine-specific dual-energy images on DECT allow for color coded display of the iodine particles inside renal lesions. With these types of images, one can differentiate a non-enhancing cyst from a solid-enhancing mass. Cysts would appear without any iodine signal because they are avascular, while solid vascular lesions demonstrate iodine as it accumulates in the volume being examined. The sensitivity of the technology for iodine signal compensates for the low vascularity of lesions like pRCC, and allow for their accurate characterization as solid masses [14, 15, 21–23]. Not only does DECT serve to differentiate renal cysts from solid masses in everyday patients, but it is also proving useful in special cases like patients with polycystic kidney disease (PCKD). In such patients, detecting solid growth enhancement in the kidney parenchyma that is replaced by a large number of cysts is very challenging. However, recent studies indicate that DECT can also be useful in detecting such growth in PCKD with reduced radiation dose; a reduction that is important considering that PCKD patients require serial imaging for follow-up for extended periods of time [24].

Along the same lines, DECT is beneficial in detecting residual cancer after thermal ablation of renal tumors. Changes, due to ablation and perinephric bleeding, may make it difficult to assess enhancement after ablative procedure, where enhancement is the sign of viable cancer. With iodine-specific dual-energy images, DECT could be a valuable technology in the urologist armamentarium to decide on the success of their therapeutic intervention. Park et al. in a recent paper, presented 47 patients treated with radiofrequency ablation (RFA) of renal tumor followed by DECT to predict tumor progression at RFA site, and he showed excellent diagnostic performance of DECT (sensitivity 100% and specificity 91.5%) for predicting local tumor progression. He then concluded that DECT allowed acceptable diagnostic performance after RFA with decreased patient radiation exposure.

Magnetic resonance imaging (MRI) and renal ultrasound are two other modalities routinely used in differentiating cystic from solid renal lesions. However, in comparison to MRI, DECT has a shorter acquisition time, is cheaper, and can be used in patients with pacemakers and other contraindications for MRI. DECT is also superior to ultrasound in that it is less operator dependent, and provides better tissue characterization.

Finally, there is a growing body of research that aims at using DECT to differentiate RCC subtypes, and to observe response to targeted therapy in metastatic RCC. These applications are based on using Iodine as a biomarker that provides additional information on tumor texture and helps us appreciate changes in tumor tissue as it responds to tyrosine kinase therapy [13, 25].

## Conclusion

DECT, if validated in clinical practice, could save patients multiple testing to differentiate between benign renal lesions and solid masses with low enhancement that could be aggressive and deadly. Future applications of this technology beyond diagnosis are being developed, and may greatly enhance the value in DECT as an imaging tool in managing patients with renal tumors.

## Consent

Written informed consent was obtained from the patient for publication of this case report and any accompanying images. A copy of the written consent is available for review by the Editor of this journal.

## Electronic supplementary material

Additional file 1: **Timeline.** (DOCX 30 KB)

## Abbreviations

CT: Computerized tomography
DECT: Dual energy computerized tomography
PCKD: Polycystic kidney disease
pRCC: Papillary renal cell carcinoma
RCC: Renal cell carcinoma
RFA: Radiofrequency ablation.
