# Supplementary material for: Low enhancing papillary renal cell carcinoma diagnosed by using dual energy computerized tomography: a case report and review of literature
Source: BMC Urol. 2014 Dec 19;14:102. doi: 10.1186/1471-2490-14-102 (PMC4289588; doi:10.1186/1471-2490-14-102)
Supplement: Supplementary file 1 — Additional file 1: Timeline. (DOCX 30 KB) [file 12894_2014_395_MOESM1_ESM.docx]

**Timeline:**

**Pathology shows papillary renal cell carcinoma**

- Presentation to Urology

- Asymptomatic and physical examination normal

Uncomplicated robotic assisted laparoscopic left partial nephrectomy

Dual energy computerized tomography showing the lesion in the left kidney to be a solid mass

**3 months**

**Size increased to 3.2 cm**

Incidental finding of 2.7 cm left lower pole renal lesion suspected of being hyper-attenuating cyst
